# Clinical Characteristics and Short-Term Outcomes of HIV Patients Admitted to an African Intensive Care Unit

**DOI:** 10.1155/2016/2610873

**Published:** 2016-10-09

**Authors:** Arthur Kwizera, Mary Nabukenya, Agaba Peter, Lameck Semogerere, Emmanuel Ayebale, Catherine Katabira, Samuel Kizito, Cecilia Nantume, Ian Clarke, Jane Nakibuuka

**Affiliations:** ^1^Department of Anaesthesia, Makerere University College of Health Sciences, Mulago National Referral Hospital, P.O. Box 7051, Kampala, Uganda; ^2^International Health Sciences University and International Hospital Kampala, Kampala, Uganda; ^3^Department of Clinical Epidemiology and Biostatistics, Makerere University College of Health Sciences, Mulago National Referral Hospital, P.O. Box 7051, Kampala, Uganda; ^4^Department of Medicine, Makerere University College of Health Sciences, Mulago National Referral Hospital, P.O. Box 7051, Kampala, Uganda

## Abstract

*Purpose*. In high-income countries, improved survival has been documented among intensive care unit (ICU) patients infected with human immune deficiency virus (HIV). There are no data from low-income country ICUs. We sought to identify clinical characteristics and survival outcomes among HIV patients in a low-income country ICU.* Materials and Methods*. A retrospective cohort study of HIV infected patients admitted to a university teaching hospital ICU in Uganda. Medical records were reviewed. Primary outcome was survival to hospital discharge. Statistical significance was predetermined in reference to *P* < 0.05.* Results*. There were 101 HIV patients. Average length of ICU stay was 4 days and ICU mortality was 57%. Mortality in non-HIV patients was 28%. Commonest admission diagnoses were Acute Respiratory Distress Syndrome (ARDS) (58.4%), multiorgan failure (20.8%), and sepsis (20.8%). The mean Acute Physiologic and Chronic Health Evaluation (APACHE II) score was 24. At multivariate analysis, APACHE II (OR 1.24 (95% CI: 1.1–1.4, *P* = 0.01)), mechanical ventilation (OR 1.14 (95% CI: 0.09–0.76, *P* = 0.01)), and ARDS (OR 4.5 (95% CI: 1.07–16.7, *P* = 0.04)) had a statistically significant association with mortality.* Conclusion*. ICU mortality of HIV patients is higher than in higher income settings and the non-HIV population. ARDS, APACHE II, and need for mechanical ventilation are significantly associated with mortality.

## 1. Introduction

Antiretroviral Therapy (ART) for the treatment of the human immunodeficiency virus (HIV) infection has been associated with improved survival among HIV infected patients all over the world [1, 2]. It is unclear in the current literature whether this progress translates into HIV infected patients admitted to the ICU [3–5]. No randomized prospective clinical trials have been conducted to guide Highly Active Antiretroviral Therapy (HAART) therapy among HIV patients in the (intensive care unit) ICU. Current evidence from some observational prospective and retrospective studies suggests that early initiation of HAART may be associated with improved outcomes among HIV patients admitted to the ICU [2, 5, 6].

Admission diagnoses and poor outcomes that were initially attributed to* Pneumocystis jirovecii* pneumonia (PJP) related respiratory failure have altered with the advent of cotrimoxazole prophylaxis [1, 7, 8]. Acute respiratory failure however has remained a leading cause of ICU admissions due to HIV-associated conditions in high-income countries [1, 7, 9].

Resource limitations in low-income countries in Africa lead to poor ICU prioritization and utilization. This leads to increased morbidity and mortality from acute illness [10–12].

HAART was introduced to Uganda in 2001. All patients have access to free HAART since 2007 accordance with local treatment guidelines. HIV care was integrated into national treatment programs in 2010 [13]. As at 2013 the national seroprevalence is currently at 7.4% [13].

Additionally, there are little or no data available regarding outcomes of HIV infected patients admitted to the ICU in HIV endemic low-income countries. Therefore, we conducted a retrospective cohort study of all HIV infected patients who had been admitted to a university teaching hospital in Kampala, Uganda. We sought to identify ICU admission diagnoses, HAART status, clinical characteristics, and outcomes, as well to compare these with and to determine predictors of survival.

## 2. Methodology

### 2.1. Study Design and Subjects

Upon obtaining a hospital ethical approval and waiver of need for informed consent, we conducted a retrospective cohort study of all HIV infected patients who had been admitted to the intensive care unit of the International Hospital Kampala (IHK) from July 2009 through 2014. IHK is an urban university teaching hospital in Kampala, Uganda, with an 11 bed ICU that can provide both respiratory and renal support services. A combined computerized and manual search of all ICU admissions identified patients with HIV who had been admitted to the ICU. All adult admissions were included.

### 2.2. Data Collection

The study team reviewed medical records using standardized pretested forms. Patients who had repeat admissions had their first admission recorded. Demographics were recorded. Admission diagnoses were classified using a predetermined list identical to that used in previous studies [3, 9]. Acute Respiratory Distress Syndrome (ARDS) was determined as the need for mechanical ventilation, absence of cardiogenic pulmonary edema with bilateral infiltrates, and a PaO_2_/FiO_2_ ratio of <200 mmHg. The Berlin definition was not in use during majority of the study period. HAART was categorized as first line, second line, and other regimens based on national treatment guidelines.

Laboratory data within three days of ICU admission (usually within 24 hours) were recorded. Organ failure status (requirement of mechanical ventilator support, renal replacement, or shock), Modified Early Warning Scores (MEWS), and Acute Physiologic and Chronic Health Evaluation (APACHE) II scores on ICU admission were also recorded.

Septic shock status was considered if patients were refractory to IV fluids and needed vasopressors to maintain mean arterial pressure (MAP) > 65 mmHg. Diagnosis of PJP was based on clinical presentation, radiographic presentations of ground-glass opacities by either computed tomography (CT) or chest radiographs (CXR). There were no facilities to perform bronchoalveolar lavage (BAL). Clinical variables included CD4 count, viral load (plasma HIV RNA if available within 6 months of admission), biochemical and hematological values especially serum albumin, need for vasopressors, and mechanical ventilation, and APACHE II score was calculated. The primary outcome was survival to hospital discharge and secondary outcomes were predictors of mortality. We also measured ICU mortality in all non-HIV patients admitted to the ICU in the study period.

### 2.3. Statistical Analysis

A statistical software package, (StataCorp. 2013.* Stata Statistical Software: Release 13*. College Station, TX: StataCorp LP) was used for analysis. Clinical characteristics are reported as means and medians or numbers and percentages. Continuous data were compared using the Student *t*-test or the Wilcoxon rank sum test, and comparisons of frequencies were made with the *χ* ^2^ test or Fisher's exact test. To describe the epidemiology of patients in the current era, we compared patient characteristics according to primary outcome and HAART status. To identify predictors of survival, we compared survivors with nonsurvivors. Unadjusted odds ratios for survival were computed for each candidate variable. Variables with *P* < 0.20 for the appropriate test were included in model-building procedures in logistic regression. Multivariate logistic regression was used to determine the most penurious variables, using all available data. Statistical significance was predetermined in reference to *P* < 0.05.

## 3. Results

### 3.1. Demographics and Clinical Characteristics

During the 5-year study period, there were 1828 patients. Of these, there were 102 HIV admissions for 101 patients. One patient was readmitted following a cardiac arrest and survived to discharge (the readmission was not included in analysis). The average age of patients was 38.13 (±16.05), and females accounted for 53.4% (Table 1). The average length of stay was 4 days (±6.16) ICU/hospital mortality was 57% (Table 2). Mortality in non-HIV ICU patients was 28% over the study period. Average APACHE II score was 24.3 (±7.2) and average MEWS was 6. Majority of patients were already diagnosed HIV patients before admission (89.01%). Of these 54% were on HAART at the time of admission with the majority (75% of those on HAART) being on first-line treatment as per national guidelines at the time (Table 2). Of patients on HAART, 43% had been on treatment for more than 6 months. Most HIV (58.5%) patients had a CD4 count less than 100, while only 4.95% patients had a viral load done at the time of admission (Table 3). Most HIV patients (68%) were on cotrimoxazole prophylaxis at admission. With regard to ICU therapy, 55.4% were on mechanical ventilation, while 36.6% were on vasopressors for septic shock (Table 4). With regard to biochemical tests, the majority had abnormal blood gases (70%), abnormal electrolytes (89.%), hypoalbuminemia (83.1%), elevated urea (72.9%), and bilirubin (54.1%). In addition, majority of patients had moderate to severe anaemia (57.3%).

### 3.2. Diagnoses of ICU Admissions

Acute respiratory failure (ARF) with ARDS was the commonest cause of admission in 59 of all 101 patients (58.2%, Table 2). Among patients with ARF, PJP and Pulmonary Tuberculosis (PTB) predominated. Respiratory infections were the commonest foci for sepsis. While sepsis was in more than 70% of patients, it was only documented in 21% of admissions as a separate illness. Of these, 36.3% needed vasopressor support for septic shock.

### 3.3. Comparison of Patients by HAART Status

Patients on HAART were significantly older (*P* = 0.01) and predominantly male (*P* = 0.02). HAART naïve patients had significantly higher incidence of toxoplasmosis as an admission diagnosis (*P* = 0.02). HAART naïve patients also had a higher incidence of PJP, although this did not reach statistical significance (*P* = 0.07).

### 3.4. Predictors of Mortality

At univariate analysis, cryptococcal meningitis (CCM), HIV encephalopathy, and ARDS were admission diagnoses associated with mortality. Acidosis, MEWS > 6, APACHE II, and Glasgow coma score (GCS) <8 were clinical characteristics associated with mortality (Table 4). Vasopressors and mechanical ventilation were treatment factors associated with mortality (Tables 5 and 6). At multivariate logistic regression analysis, only having mechanical ventilation, high APACHE II and ARDS remained statistically significant (Table 6).

We assessed the goodness of fit of our final model using the Hosmer and Lemeshow statistic and the corresponding chi-square value is 124.41, *P* = 0.258. This showed a good model.

## 4. Discussion

We conducted this study in a period where there is affordable access to HIV diagnosis and medication in addition to cotrimoxazole prophylaxis in low resource settings. We believe that this study may be the first of its kind looking at HIV in the ICU in an African setting.

The study was conducted in a university teaching hospital with the ability and protocols to provide basic evidence based critical care support such as low tidal volume ventilation; early sepsis recognition and treatment; prone positioning for ARDS, albeit in a low-income country.

The study demographics such as age and sex were similar to that reported in the literature in similar settings [10]. Additionally, this age group is similar to that in which HIV infection is highest in our study setting [14].

The HIV endemicity of our setting is manifested by the high rate of admission (102 in 60 months) for a private medical facility compared to the developed world where admission rates are lower in subsidized care [1, 3, 14]. The commonest admission diagnoses were similar to that reported in the literature. Acute respiratory failure was the commonest reason for admission and as reported in previous studies, infectious causes such as PJP, PTB, and community acquired pneumonia were predominant causes.

Causes of ICU admissions observed in our study are not different from that observed in studies conducted elsewhere regardless of economic status [7, 15, 16]. In our study, majority of patients were known HIV patients to the healthcare system. The fact that 11% were undiagnosed HIV patients at ICU admission also points to the efficiency of the national voluntary testing program in our country. More than 50% of all admissions were external referrals. This points to the dearth of ICU beds in the country [10].

Mortality among HIV patients in our setting was higher than in the non-HIV population. A possible explanation is that the severity of illness is worse among HIV patients. Prognostic factors of mortality for HIV patients admitted to the ICU include CD4 count; APACHE II score; features of organ failure (evidenced by the need for mechanical ventilation, renal replacement therapy, and vasopressor support); and hypoalbuminemia. These were identified in studies conducted in North America, Latin America, and Europe [7, 14–16].

More than half the patients presented with CD4 counts <100 × 10^9^/L. This would also explain the high degree of opportunistic infections seen in these patients. At univariate analysis CD4 was associated with mortality; however after adjusting it was nonsignificant. This was observed in other studies [2, 14, 17, 18]. That viral load testing was not routinely done because of high costs of testing. The median CD4 count of 63 × 10^9^/L was similar to that reported in some literature [3, 19]. The threshold for initiating HAART in our country is lower than in higher income settings (our country guidelines recommended starting at a CD4 count of 250 × 10^9^/L or less at the time compared with 500 × 10^9^/L in high-income countries); this may explain the higher severity of illness scores that our patients presented with. However, unlike in the same literature it was not associated with mortality at multivariable analysis.

The APACHE II score in our study was higher than that reported in other studies [5, 14, 16]. This may also reflect delay in decision to ICU admission or quick deterioration of health. The median APACHE of 25 predicted >50% mortality. This was true in our study, at bivariate analysis, persisting even after adjusting for multiple variables.

With regard to serum albumin levels, our study did not detect any significant difference or relationship with mortality as was reported in the previous literature [1]. This is possibly due to the low number of patients in whom serum albumin measurement was recorded.

Organ failure was manifest by the need for mechanical ventilation, which was in all patients who presented with respiratory failure. This is similar to other studies where respiratory failure was also the primary reason for admission [1, 3, 20]. An interesting finding is that all patients who presented with acute respiratory failure got mechanical ventilation and suffered ARDS. They were also 4.5 times more likely to die. The need for mechanical ventilation was associated with mortality at multivariate analysis. Less significant, however, was the need for vasopressor support in patients with septic shock. In this study the foci of sepsis seemed to be of pulmonary origin. This is similar to what has been reported in the literature.

Severe sepsis has been documented as a significant predictor of mortality among HIV patients in other studies [21]. And while sepsis is the most important risk factor for mortality in HIV/AIDS patients admitted to ICUs, affecting short- and longer-term survival [18], we were unable to demonstrate this at multivariate analysis.

HIV/AIDS variables, such as CD4 T-cell count and HIV RNA level, seem to play a secondary role for the prognosis of critically ill HIV/AIDS patients [4]. The mechanisms by which this happens are not clearly understood. A study in Brazil highlighted the occurrence of nosocomial severe infections and the great impact of sepsis on 1- and 6-month survival and confirmed that CD4 the T-cell count, the HIV RNA level, and other HIV/AIDS variables were not predictive of 30-day and 6-month outcomes [21].

While not our primary outcome, our study failed to demonstrate that being on HAART at admission was superior to being HAART naïve. This may have been due to the retrospective nature of our study; low study power; genetic factors; and drug related issues (e.g., bioavailability). However, we noted a weak link between being HAART naïve and development of PJP. Another limitation was that we were unable to document in-ICU HAART initiation due to archival reasons. Early HAART initiation and albumin replacement in HIV infected ICU patients were not standards of care during the study period. There is still the fear that early initiation in PTB, CCM, and Kaposi sarcoma (KS) may predispose patients to immune reconstitution syndrome (IRIS), although it may be argued that the ICU is the best place to manage a patient with IRIS. Demonstrating the impact of HAART on outcomes in our study population has been limited by the short treatment durations majority of patients were under. Additionally, we were unable to document immunologic response and adherence to HAART. Our study had limitations due to its retrospective nature. We were unable to obtain a number of clinical data due to archival reasons.

## 5. Conclusion

Our study is the first study in Africa to show that ICU mortality due to HIV infection is higher than in higher income countries and higher than that seen in non-HIV ICU patients. A high APACHE II, ARDS, and being on mechanical ventilation are strong predictors of mortality. Our study demonstrated no benefit of HAART status at admission on mortality outcomes.

## Figures and Tables

**Table 1:** Baseline patient characteristics.

Variable	*N*	Categories	Number	Percentage
Sex	101	Male	47	46.53
		Female	54	53.47

Time since diagnosis	101	Known HIV positive	90	89.01
		Made at ICU admission	11	10.99

CD4 range	101	More than 300	12	11.88
		100 to <300	30	29.70
		Less than 100	59	58.42

HAART status	101	On HAART	56	55.45
		HAART naive	45	44.55

HAART regimen	101	1st line	37	36.63
		2nd line	12	11.88
		Not known	7	6.93
		Naïve	45	44.55

Cotrimoxazole status	101	On cotrimoxazole	69	68.32

Survival	101	Discharged	44	43.56
		Died	57	56.44

Mechanical ventilation	101	Yes	57	55.44

Vasopressors for septic shock	101	Yes	37	36.63

**Table 2:** Univariate analysis of admission diagnoses.

Admission diagnosis	*N* (%)	Survived	Died	*P* value
HIV encephalopathy	3 (2.97)	3 (6.82)	0 (0)	0.079
Kaposi sarcoma	8 (7.92)	4 (9.09)	4 (7.02)	0.702
Hypotension	22 (21.78)	7 (15.91)	15 (26.32)	0.209
Gastrointestinal	5 (4.95)	2 (4.55)	3 (5.26)	0.869
Multiorgan	28 (27.72)	11 (25.00)	17 (29.82)	0.591
Liver	6 (5.94)	2 (4.55)	4 (7.02)	0.694
Anemia	18 (17.82)	8 (18.18)	10 (17.54)	0.934
PTB	18 (17.82)	6 (13.64)	12 (21.05)	0.334
Toxoplasmosis	14 (3.96)	2 (4.55)	12 (21.05)	0.791
CCM	8 (7.92)	6 (13.64)	2 (3.51)	**0.076**
Meningitis	4 (3.96)	1 (2.27)	3 (5.26)	0.630
ARF	59 (58.42)	16 (36.36)	43 (75.44)	**0.000**
AKI	20 (19.80)	9 (20.45)	11 (19.30)	0.885
Sepsis	21 (20.79)	7 (15.91)	14 (24.56)	0.288
PJP	21 (20.79)	8 (18.18)	13 (22.81)	0.570

PTB: pulmonary tuberculosis, ARF: acute respiratory failure, AKI: acute kidney injury, PJP: *Pneumocystis jirovecii* pneumonia, and CCM: cryptococcal meningitis.

**Table 3:** Baseline clinical and biochemical characteristics at admission.

Variable	Combined		Survived		Died		*P*
	*n*	Mean ± SD	*n*	Mean ± SD	*n*	Mean ± SD	
Age	101	38.13 ± 16.05	44	37.38 ± 17.23	57	38.70 ± 15.20	0.683
CD4	79	139 ± 189	35	160 ± 206	44	123 ± 175	0.393
Urea	74	61.30 ± 65.93	36	65.42 ± 80.39	38	57.41 ± 49.32	0.605
Creatinine	75	2.33 ± 3.05	37	2.21 ± 3.06	38	2.44 ± 3.08	0.750
Urea: Crea	74	37.98 ± 29.57	36	37.69 ± 25.05	38	38.27 ± 33.63	0.933
Albumin	52	21.47 ± 10.77	25	22.34 ± 12.47	27	20.67 ± 9.09	0.582
Hemoglobin	82	9.61 ± 3.03	40	9.58 ± 3.59	42	9.63 ± 2.42	0.944
^*∗*^Metabolic acidosis	**54**	**7.31 ± 0.24**	**25**	**7.43 ± 0.14**	**29**	**7.20 ± 0.26**	**0.0004**
MEWS	**101**	**6.35 ± 3.18**	**44**	**4.80 ± 2.87**	**57**	**7.54 ± 2.90**	**0.000**
APACHE II	**101**	**24.32 ± 7.23**	**44**	**20.07 ± 5.97**	**57**	**27.60 ± 6.39**	**0.000**
GCS	**101**	** 10.52** ** ± 4.63**	**44**	**12.41 ± 3.73**	**57**	**9.07 ± 4.77**	**0.0002**
Days in ICU	101	4.00 ± 5.58	44	4.57 ± 6.16	57	3.56 ± 5.11	0.372

^*∗*^Metabolic acidosis pH < 7.15; MEWS: modified early warning score; APACHE: acute physiologic and chronic health evaluation.

**Table 4:** Univariate analysis of key characteristics by survival outcome.

Variable	Categories	Combined	Survived	Died	*P*
Sex	Male	47 (46.53)	19 (43.18)	28 (49.12)	0.55
	Female	54 (53.47)	25 (56.82)	29 (50.88)	

CD4 range	Above 300	12 (11.88)	7 (15.91)	5 (8.77)	**0.02**
	100 to 300	30 (29.70)	18 (40.91)	12 (21.05)	
	Below 100	57 (58.42)	19 (43.18)	40 (70.18)	

HAART	On HAART	56 (55.45)	23 (52.27)	33 (57.89)	0.57
	HAART naive	45 (44.55)	21 (47.73)	24 (42.11)	

ARV regimen	1st line	37 (36.63)	14 (31.82)	23 (40.35)	0.49
	2nd line	12 (11.88)	7 (15.91)	5 (8.77)	
	Not known	7 (6.93)	2 (4.55)	5 (8.77)	
	Naive	45 (44.55)	21 (47.73)	24 (42.11)	

Cotrimoxazole	Yes	69 (68.32)	30 (68.18)	39 (68.42)	0.98
	No	32 (31.68)	14 (31.82)	18 (31.58)	

Abnormal electrolytes	Yes	11 (10.89)	6 (13.64)	5 (8.77)	0.52
	No	90 (89.11)	38 (86.36)	52 (91.23)	

Time on HAART	Not known	62 (61.39)	29 (65.91)	33 (57.89)	0.42
	Less than 1 month	15 (14.85)	7 (15.91)	8 (14.04)	
	1 to 6 months	7 (6.93)	1 (2.27)	6 (10.53)	
	Beyond 6 months	17 (16.83)	7 (15.91)	10 (17.54)	

Septic shock	Yes	37 (36.63)	7 (15.91)	30 (52.63)	**0.00**
	No	64 (63.37)	37 (84.09)	27 (47.37)	

Ventilator	Yes	57 (56.44)	12 (27.27)	45 (78.95)	**0.00**
	No	44 (43.56)	32 (72.73)	12 (21.05)	

Urea	Low (<8 mg/dL)	1 (1.35)	1 (2.78)	0 (0)	0.58
	Normal	19 (25.68)	9 (25.00)	10 (26.32)	
	High (>20 mg/dL)	54 (72.97)	26 (72.22)	28 (73.68)	

Creatinine	Low (<50 *μ*mol/L)	36 (48.00)	20 (54.05)	16 (42.11)	0.41
	Normal	3 (4.00)	2 (5.41)	1 (2.63)	
	High (>150 *μ*mol/L)	36 (48.00)	15 (40.54)	21 (55.26)	

Albumin	Low (<30 mg/dL)	44 (83.02)	20 (76.92)	24 (88.83)	0.29
	Normal	9 (16.98)	6 (23.08)	3 (11.11)	

Hemoglobin	Normal	13 (15.85)	7 (17.50)	6 (14.29)	0.85
	Mild anemia	22 (26.83)	10 (25.00)	12 (28.57)	
	Moderate anemia	23 (28.05)	10 (25.00)	13 (30.95)	
	Severe anemia	24 (29.27)	13 (32.50)	11 (26.19)	

**Table 5:** Comparison of patients by HAART status.

Variable	Categories	HAART	Naive	*P*
*Mean age (years) ± SD*		*41.9 ± 15.3*	*33.4 ± 16.0*	*0.01*

CD4 ± SD		144.5 ± 174.6	133.1 ± 209.7	0.80

APACHE ± SD		23.7 ± 7.4	25.1 ± 7.0	0.35

GCS ± SD		10.2 ± 4.5	10.9 ± 4.8	0.48

Length of hospital stay		3.8 ± 6.2	4.2 ± 4.7	0.74

Gender	*Male*	*32 (68.1)*	*15 (31.9)*	* 0.02*
	Female	24 (44.4)	30 (55.6)	

Outcome	Died	33 (57.9)	24 (42.1)	0.57
	Survived	23 (52.3)	21 (47.7)	

Diagnosis	HIV encephalopathy	2 (66.7)	1 (33.3)	0.69
	No	54 (55.1)	44 (44.9)	
	Kaposi sarcoma	6 (75.0)	2 (25.0)	0.25
	No	50 (53.8)	43 (46.2)	
	PTB	8 (44.4)	10 (55.6)	0.30
	No PTB	48 (57.8)	35 (42.2)	
	*Toxoplasmosis*	*0 (0)*	*14 (100)*	* 0.02*
	No toxo	56 (57.7)	41 (42.3)	
	CCM	3 (37.5)	5 (62.5)	0.29
	No CCM	53 (57.0)	40 (43.0)	
	ARDS	34 (57.6)	25 (42.4)	0.60
	No ARDS	22 (52.4)	20 (47.6)	
	Sepsis	12 (57.1)	9 (42.9)	0.86
	No sepsis	44 (55.0)	36 (45.0)	
	AKI	13 (65.0)	7 (35.0)	0.34
	No AKI	43 (53.1)	38 (46.9)	
	PCP	8 (38.1)	13 (61.9)	0.07
	No PCP	48 (60.0)	32 (40.0)	

**Table 6:** Multivariate analysis of predictors of mortality.

Variable	Categories	OR	95% CI	*P* value	Adjusted OR	95% CI	*P* value
Interventions	Vasopressors	**0.17**	**0.07–0.44**	0.000^**∗**^	0.51	0.12–2.14	0.351
	Mechanical ventilation	**1.30**	**0.04–0.25**	0.000^**∗**^	**1.14**	**0.09–0.76**	**0.006**

GCS		**0.84**	**0.76–0.93**	0.001^**∗**^	0.95	0.80–1.13	0.560

APACHE II		**1.20**	**1.11–1.30**	0.000^**∗**^	**1.24**	**1.10–1.40**	**0.000**

MEWS		**1.39**	**1.18–1.63**	0.000^**∗**^	1.07	0.86–1.35	0.543

^*∗*^Metabolic acidosis		**0.002**	**0.00–0.15**	0.005^**∗**^			

Disease	CCM	**4.34**	**0.83–22.67**	0.082	8.15	0.33–202.1	0.200
	ARDS	**0.14**	**0.05–0.40**	0.000^**∗**^	**4.5**	**1.07–16.7**	**0.039**

^*∗*^Metabolic acidosis pH < 7.15; APACHE: acute physiologic and chronic health evaluation.
